# Antimicrobial Activity of Chitosan Oligosaccharides with Special Attention to Antiparasitic Potential

**DOI:** 10.3390/md19020110

**Published:** 2021-02-12

**Authors:** Nayara Sousa da Silva, Nathália Kelly Araújo, Alessandra Daniele-Silva, Johny Wysllas de Freitas Oliveira, Júlia Maria de Medeiros, Renata Mendonça Araújo, Leandro De Santis Ferreira, Hugo Alexandre Oliveira Rocha, Arnóbio Antônio Silva-Junior, Marcelo Sousa Silva, Matheus de Freitas Fernandes-Pedrosa

**Affiliations:** 1 Postgraduate Program in Pharmacy, Faculty of Pharmacy, Federal University of Rio Grande do Norte, Natal 59012-570, Brazil; nay.sou@hotmail.com; 2 Department of Pharmacy, Faculty of Pharmacy, Federal University of Rio Grande do Norte, Natal 59012-570, Brazil; nakar_rn@hotmail.com (N.K.A.); lean_sf@yahoo.com.br (L.D.S.F.); arnobiosilva@gmail.com (A.A.S.-J.); 3 Postgraduate Program in Development and Technological Innovation in Medicines, Bioscience Center, Federal University of Rio Grande do Norte, Natal 59072-970, Brazil; alessandra.daniele@outlook.com; 4 Postgraduate Program in Biochemistry, Bioscience Center, Federal University of Rio Grande do Norte, Natal 59072-970, Brazil; johny3355@hotmail.com; 5 Postgraduate Program in Chemical Engineering, Technology Center, Federal University of Rio Grande do Norte, Natal 59072-970, Brazil; medeirosjuulia@gmail.com; 6 Chemistry Institute, Federal University of Rio Grande do Norte, Natal 59072-970, Brazil; renat.onca@gmail.com; 7 Department of Biochemistry, Bioscience Center, Federal University of Rio Grande do Norte, Natal 59072-970, Brazil; hugo-alexandre@uol.com.br; 8 Department of Clinical and Toxicological Analysis, Faculty of Pharmacy, Federal University of Rio Grande do Norte, Natal 59012-570, Brazil; mssilva.ufrn@gmail.com; 9 Global Health and Tropical Medicine, Institute of Hygiene and Tropical Medicine, University of Nova Lisboa, 1099-085 Lisbon, Portugal

**Keywords:** antiprotozoal, chitooligosaccharides, neglected tropical disease

## Abstract

The global rise of infectious disease outbreaks and the progression of microbial resistance reinforce the importance of researching new biomolecules. Obtained from the hydrolysis of chitosan, chitooligosaccharides (COSs) have demonstrated several biological properties, including antimicrobial, and greater advantage over chitosan due to their higher solubility and lower viscosity. Despite the evidence of the biotechnological potential of COSs, their effects on trypanosomatids are still scarce. The objectives of this study were the enzymatic production, characterization, and in vitro evaluation of the cytotoxic, antibacterial, antifungal, and antiparasitic effects of COSs. NMR and mass spectrometry analyses indicated the presence of a mixture with 81% deacetylated COS and acetylated hexamers. COSs demonstrated no evidence of cytotoxicity upon 2 mg/mL. In addition, COSs showed interesting activity against bacteria and yeasts and a time-dependent parasitic inhibition. Scanning electron microscopy images indicated a parasite aggregation ability of COSs. Thus, the broad biological effect of COSs makes them a promising molecule for the biomedical industry.

## 1. Introduction

The advance of antimicrobial resistance and the climate crisis outline an alarming scenario. Antimicrobial resistance is diminishing the treatment options for microbial diseases, such as bacteria, yeast, and parasites, leading to a post-antibiotic era [1,2]. In addition, global warming can directly modify infectious diseases by affecting the pathogen, the host-vector relation, and the transmission environment. Thus, it would lead to a geographical expansion of vector-borne diseases, as well as a reduction in human immunity [3,4]

A climatic suitability model study suggests an increased number of leishmaniases vector species in Europe under future climatic conditions [5]. A rise in temperature would also prompt a shorter life cycle of *Trypanosoma cruzi*, which impacts the transmission of Chagas disease [6]. Cases of Chagas disease have been registered in non-endemic areas, such as European countries, the United States, and Canada, as a result of migratory flows [7,8].

Leishmaniasis and Chagas disease are neglected tropical diseases (NTDs) caused by *Trypanosoma cruzi* and *Leishmania* sp., respectively, protozoa from the Trypanosomatidae family [9]. Currently, *T. cruzi* is endemic in 21 Latin American countries and infects 6 to 7 million people [10]. Likewise, leishmaniasis is endemic in 18 Pan-American countries [11]. Both conditions are serious health problems leading to substantial morbidity and mortality. However, the available treatments for these parasitic diseases exhibit high rates of adverse effects and toxicity, thus highlighting the importance and urgency of new therapy options [12,13,14,15].

In this scenario, chitin is a natural polysaccharide commercially obtained from the shells of sea arthropods. Annually, from the seafood processing industry, 6 to 8 million tons of shell waste are generated globally; however, most of it is incorrectly discarded in nature [16]. Chitosan is an *N*-acetyl-D-glucosamine (GlcNAc) and D-glucosamine (GlcN) copolymer obtained from the partial deacetylation of chitin. Chitosan is biocompatible, nontoxic, and also has biodegradable proprieties, hence it is broadly studied in many fields [17]. It has been explored for its antibacterial activity against Gram-positive and Gram-negative bacteria [18,19]. Different mechanisms have been proposed for the antimicrobial activity of chitosan, such as the perturbation of ion transport on the microorganisms [19,20]. Additionally, the cationic nature of chitosan leads to an augmented bioadhesion in the microorganism’s membranes, such as fungal membranes, for example [20]. We believe that this property of chitosan can also be explored against parasites from the Trypanosomatidae family. However, a wide range of variables involved with this polysaccharide, such as high molecular mass, deacetylation degree, the pattern of acetylation, and mainly its solubility character, remain as the main barriers to its broad use in the biomedical area. Thus, we hypothesized that it is possible to tailor these limitations by preparing small and soluble chitooligosaccharides (COSs), which can be designed and have their properties controlled for this purpose.

Previous studies have shown the antimicrobial [21], anti-inflammatory [22], antitumoral [23], and antioxidant [24] activities of COSs, as well their potential use in Alzheimer disease [25]. More details on the biological properties and applications of COSs are described elsewhere [26,27]. Recently, COSs have been exploited in nanotechnology aiming to tackle microbial resistance [28], and also tumor imaging and therapy [29] and drug-delivery [30]. The structural characteristic of COSs regarding their action mechanisms, such as degree of deacetylation (DD), degree of polymerization (DP), the pattern of acetylation (PA), and molecular weight (MW), are direct outcomes from the type of hydrolysis performed, such as chemical, physical or enzymatic hydrolysis [24,31]. In our recently published paper [32], the performance of *Bacillus toyonensis* chitosanase was greater in COS production than other commercial enzymes after immobilization, evidencing the stability of this enzyme, in a prospect to their use at industrial scale.

Hence, the search for new molecules with activity against *T. cruzi* and *Leishmania* sp. that are effective and with fewer adverse events is emphasized. Once, only a few studies described the effect of COSs against parasites from the Trypanosomatidae family [33]; this fact, therefore, stimulated our investigation of the trypanocidal activity of these oligomers. This study reports the enzymatic hydrolysis of chitosan to produce a COS mixture using chitosanases obtained from *B. toyonensis*, identified and isolated earlier by our research group [34]. Following the evaluation of the broad antimicrobial effects of COSs on bacteria, yeast, and trypanosomatids *T. cruzi* and *L. amazonensis* in vitro, as well the morphological alterations on these parasites caused by COSs incubation, we aimed to confirm our hypothesis about the biological potential of these oligomers. The COSs produced were characterized by 1D and 2D NMR and mass spectroscopy. Our results indicate a broad antimicrobial action of COS mixture, with an interesting time-dependent action upon both parasites. To our knowledge, this is the first time that the effects of COSs upon trypanomatids with SEM images are described. The possible transformation of a sea-food processing residue into a molecule of biotechnological interest with pharmacological activities results in a positive ecological, economic and biomedical impact.

## 2. Results

### 2.1. COS Production and Analysis

COSs were generated by the enzymatic hydrolysis of chitosan using *B. toyonensis* chitosanase. To elucidate the structural characteristics of COS, the oligosaccharide was analyzed by NMR and mass spectrometry.

#### 2.1.1. Nuclear Magnetic Resonance Spectroscopy

The structural elucidation of COSs was established by the interpretation of uni- (^1^H and ^13^C-DEPTq) and bidimensional (^1^H-^1^H COSY, ^1^H-^13^C HSQC, and ^1^H-^13^C HMBC) NMR analysis. Data demonstrated the presence of a mixture of COSs with the predominance of GlcN over GlcNAc. A deacetylation degree of 81.14% was determined by applying the integration values in Equation (1) [35].

**Figure 1:**
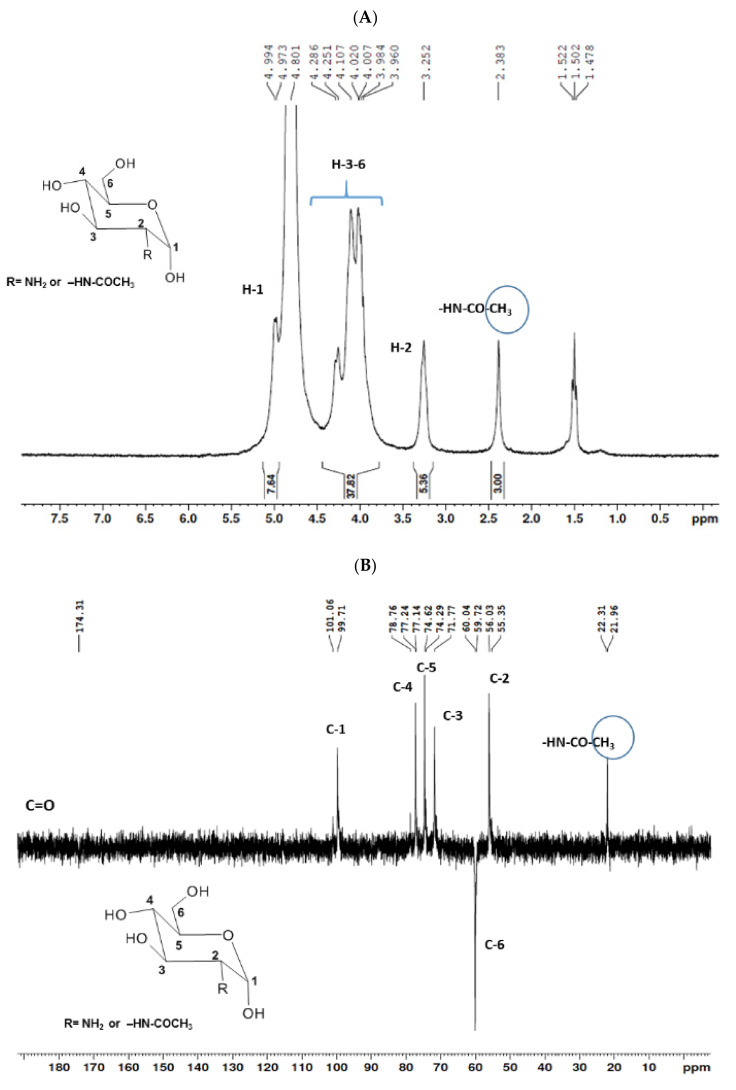
Unidimensional NMR spectra of chitooligosaccharides (COSs) (D_2_O, 300 MHz). (**A**) ^1^H spectrum; (**B**) ^13^C spectrum.

**Figure 2:**
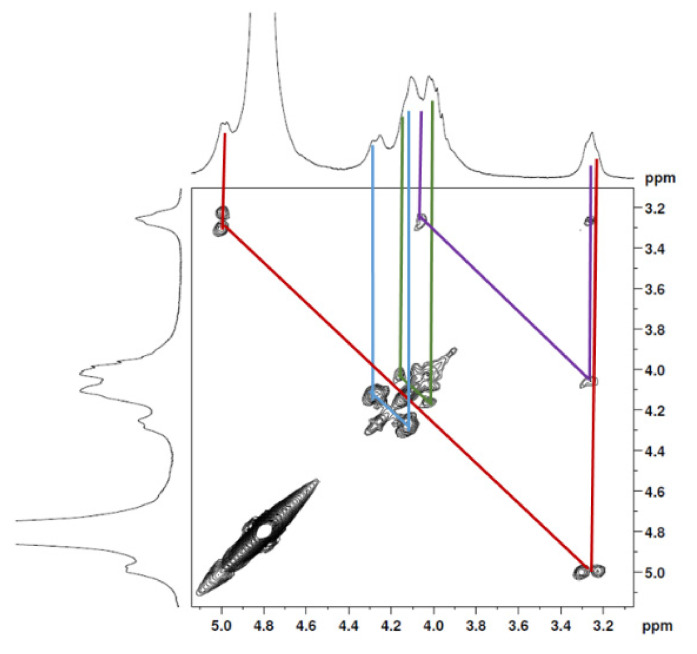
COSY 2D NMR spectrum of COS (D_2_O, 300 MHz).

**Figure 3:**
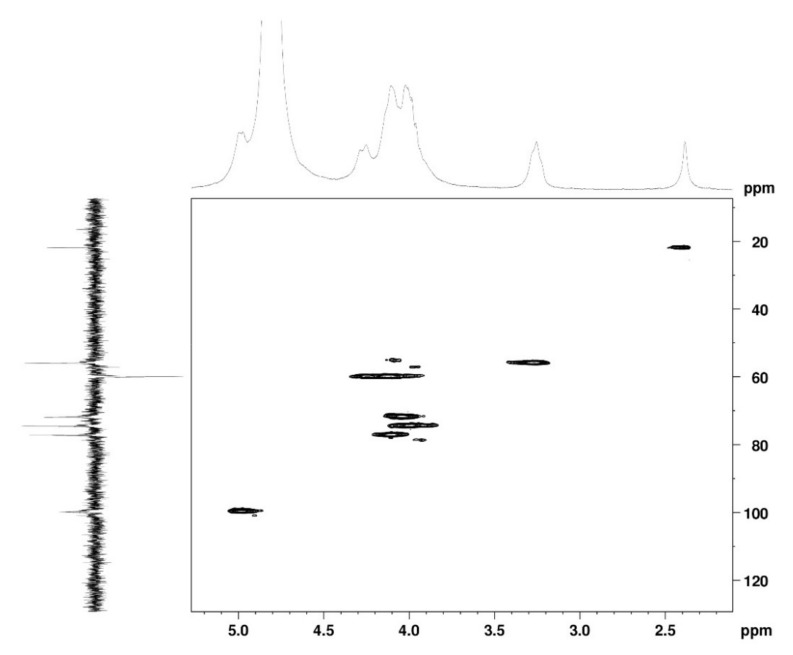
HSQC 2D NMR spectrum of COS (D_2_O, 300 MHz).

**Figure 4:**
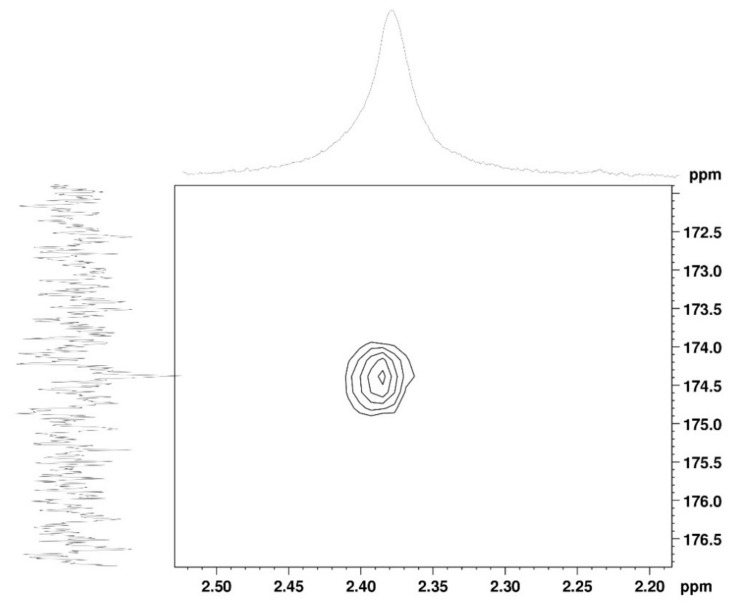
HMBC 2D NMR spectrum of COS (D_2_O, 300 MHz).

The ^1^H NMR spectrum (Figure 1A) displayed signals between δ_H_ 3.2–4.3 assigned to the H_2-6_ from GlcN and GlcNAc monomers. Furthermore, the duplet on δ_H_ 4.99 (*J* = 6.3 Hz) was characteristic of the H_1α_ anomeric hydrogen of COSs with β-1-4 glycosidic bonds [36]. The DEPTq-^13^C NMR spectrum (Figure 1B) showed overlapping spectral lines between δ_C_ 99.8 and 101.0, characteristic of anomeric carbons and signals between δ_C_ 71.0 and 79.0 compatible with oxymethine C_3_, C_4_, and C_5_ carbons from the β-GlcN and β-GlcNAc units, whereas spectra lines with inverted amplitude at δ_C_ 59.7–60.0 were assigned to methylene C_6_ carbons. This spectrum also permitted the identification of C_2_–N carbons in δ_C_ 56.0 and 55.4 and the methyl carbon from GlcNAc monomers at δ_C_ 21.9 [36,37]. The ^1^H-^1^H-COSY (Figure 2) revealed sequential connectivity of the hydrogens H_1_ (δ_H_ 4.99) and H_2_ (δ_H_ 3.25), H_2_ and H_3_ (δ_H_ 4.0), and H_5_ (δ_H_ 4.10) with the diastereotopic hydrogens 2H_6_ (δ_H_ 4.28 and 4.05) from the GlcN units, the majority monomer. Due to the overlap of the contour diagram of GlcN on COSY, it was not possible to correlate the data from the GlcNAc unit. Further analysis of the correlations observed in the HSQC spectrum (Figure 3) confirmed the attribution of each hydrogen to the respective carbon and ratified the presence of β-GlcN and β-GlcNAc units in the COS mixture [38,39]. The correlation observed in the HMBC spectrum (Figure 4) of the methyl hydrogens at δ_H_ 2.38 to carbonyl carbon at δ_C_ 174.4 (^2^*J*_CH_) confirmed the presence of an acetyl group of the GlcNAc units.

#### 2.1.2. Mass Spectrometry

**Figure 5:**
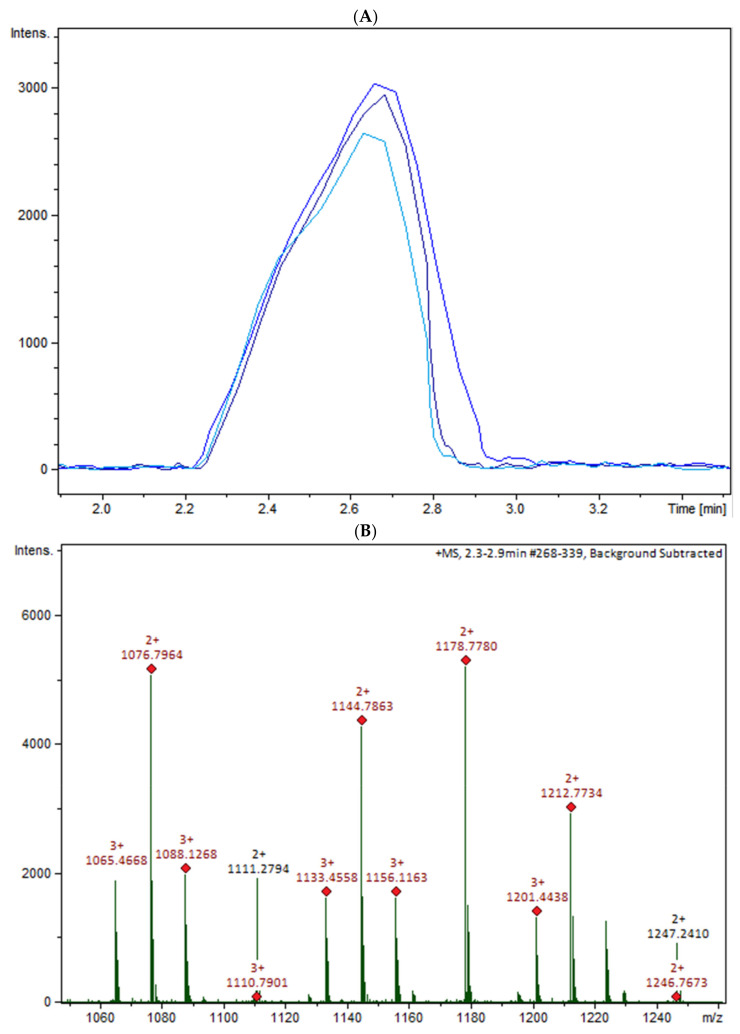

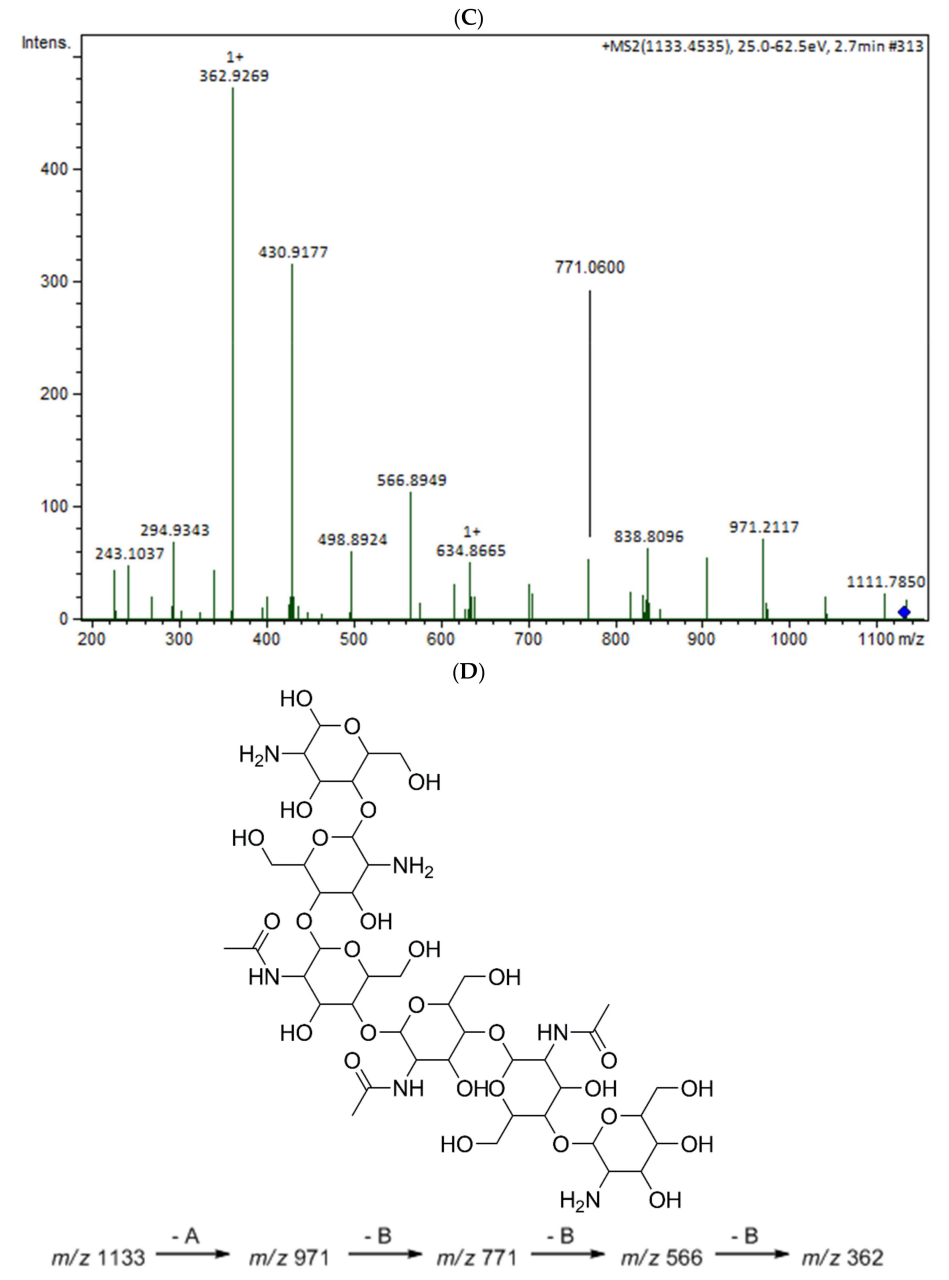
(**A**) HPLC-MS/MS chromatogram of the ion *m*/*z* 1133 in the fractions COSG1 (dark blue), COSG2 (blue), and COSG3 (light blue); (**B**) MS spectrum between *m*/*z* 1050 to 1250 of the peaks of 2.3 to 2.9 min from COSG3; (**C**) MS/MS from the ion m/z 1133 fragmentation of COSG3; (**D**) Proposed structure of hexamer m/z 1133 and fragmentation (A: GlcN and B: GlcNAc).

The oligomers in the COS mixture were identified by HPLC-MS/MS (ESI-QTOF). The non-hydrolyzed chitosan was removed by gel permeation chromatography (GPC), and three fractions were collected (COSG1, COSG2, and COSG3) for the HPLC-MS/MS. This pre-purification process allowed a lower suppression effect noted initially for the reaction sample, and also decreased the viscosity, which was necessary especially for the chromatographic system and spray formation. As demonstrated in Figure S1, the oligomers co-eluted approximately between 2.2 and 2.9 min, and three small peaks were also noted around 19 min in Figure S2. Besides low yield, it was not possible to separate or isolate the oligomers. However, all fractions showed a peak near 2.5 min with the presence of the ion *m*/*z* 1133 (Figure 5A). Figure 5B shows the COSG3 mass spectra of the peak at 2.5 min and ions from *m*/*z* 1050 to 1250. The ion *m*/*z* 1133 was identified as a hexamer with three acetylation moieties. The MS/MS spectrum for *m*/*z* 1133 (Figure 5C) allowed the presumption that the structure was an acetylated hexamer (Figure 5D). The hexamer in one extremity was composed of two units of GlcN followed by three GlcNAc units, and, at the other extremity, GlcN. This sequence was proposed by the loss of GlcN/GlcNAc units generating ions *m*/*z* 971, 771, 566, and 362 ion fragmentation from the *m*/*z* 1133, as a common fragmentation pathway for saccharides [40].

### 2.2. Biocompatibility Assay

**Figure 6:**
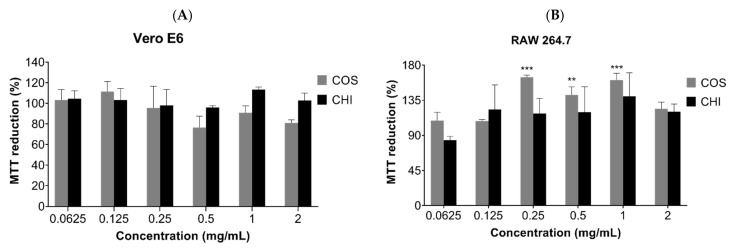
MTT reduction after 24h of cell incubation with COS5, COS, and chitosan (CHI) (0.0625–2 mg/mL) or Dulbecco’s Modified Eagle Medium (DMEM) medium supplemented with 10% FBS (*v*/*v*). (**A**) Vero E6 cells; (**B**) RAW 264.7 cells. Values are expressed as mean ± SD (*n* = 3). ** *p* < 0.01, and *** *p* < 0.001, compared to the control group (100% viability).

MTT assay was used to evaluate the cytotoxic effect of COSs and chitosan (CHI) on kidney epithelial cells of the African green monkey (Vero E6, ATCC CRL-1586) and murine macrophages cell lines (RAW 267.4, ATCC TIB-71). After 24 h incubation, there was no indication of chitosan or oligomer toxicity in the tested cell lines (Figure 6). The incubation of COSs with RAW cells exhibited a rise in the MTT reduction of 64.5% + 2.6, 42% + 10, and 60.7% + 8.9 at the concentrations of 0.25 mg/mL, 0.5 mg/mL, and 1 mg/mL, respectively, which suggests an increase in the metabolism or in cellular proliferation (Figure 6B).

### 2.3. The Antimicrobial Activity of COSs

**Figure 7:**
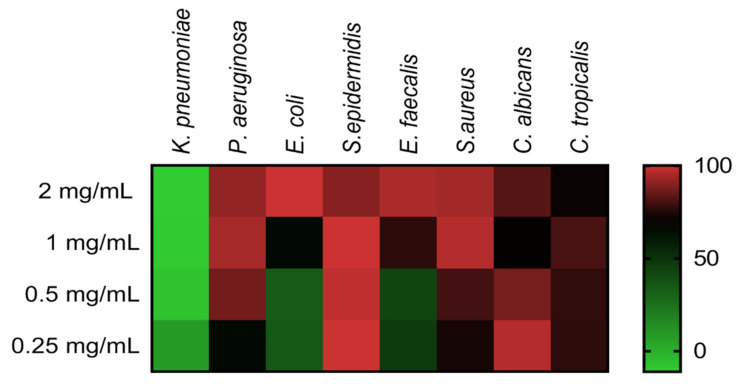
Heat map of different microorganisms’ growth inhibition after COS treatment for 24 h. Results are expressed as the mean percentage of inhibition compared to the positive group of each microorganism.

Minimal inhibitory concentration (MIC) evaluation of COSs was performed employing ATCC bacteria and yeast in the microdilution method. Figure 7 shows the heat map with the percentage of growth inhibition of COS concentrations on microorganisms. The heat map evidenced the concentration-response effect of COSs, with notable inhibition even below the MIC values (Table 1). COSs exhibited a higher antimicrobial effect upon Gram-positive bacteria and *Candida* yeast; MIC for *S. epidermidis* and *C. albicans* were <0.25 and 0.5 mg/mL, respectively. Interestingly, 1 mg/mL of COS on *C. albicans* and 2 mg/mL on *C. tropicalis* had a lower inhibition rate than the inferior concentration of the same oligomer in these yeasts.

### 2.4. Antiparasitic Activity of COSs

**Figure 8:**
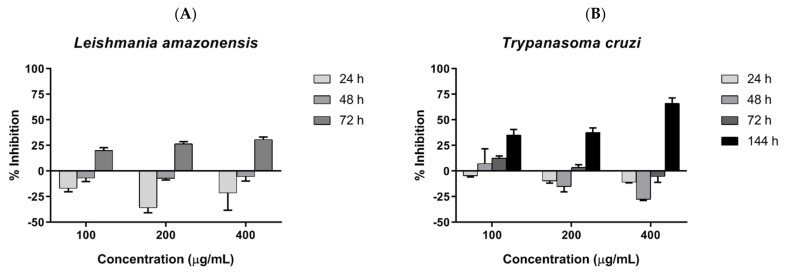
(**A**) COSs’ effects on promastigotes of *L. amazonensis* after different incubation times (**B**) COSs’ effects on epimastigote forms of *T. cruzi* after different incubation times. The values are expressed as the mean percentage of inhibition ±SEM (*n* = 3).

Parasite inhibition was investigated by incubating COSs with epimastigote forms of *T. cruzi* and promastigote forms of *L. amazonensis*, and assessed by resazurin reduction. COSs demonstrated similar outcomes in both tested trypanosomatids. The groups treated with COSs showed a greater reduction in resazurin than the control group, suggesting a proliferation of the parasite. However, a shift in parasite response to COSs occurred after 72 h, with the decrease in resazurin indicating parasite death. Therefore, the chitosan oligomers displayed a time-dependent effect on the tested parasites. The concentration of 400 μg/mL exhibited greater inhibition in both parasites: 30.79% ± 2.51 against *L. amazonensis* after 72 h (Figure 8A), and 66.18% ± 5.27 against *T. cruzi* after 144 h (Figure 8B).

**Figure 9:**
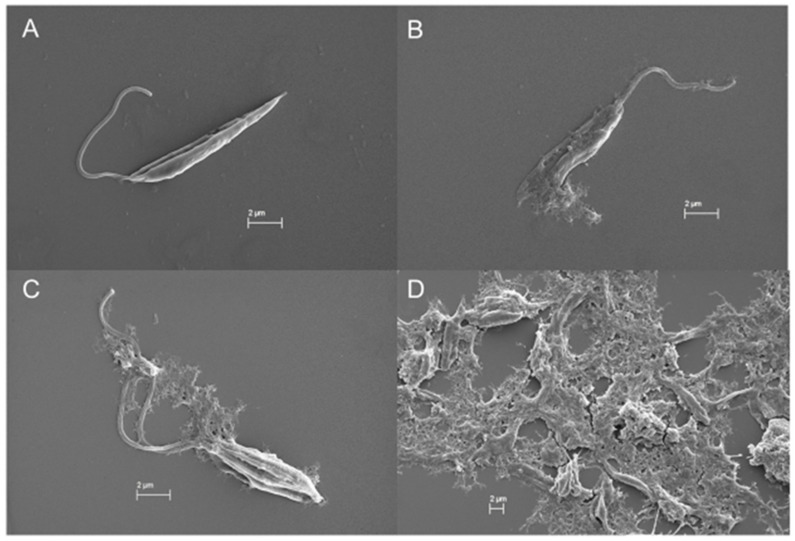
SEM images of (**A**) promastigote forms of *L. amazonensis*; (**B**–**D**) promastigote treated with 400 μg/mL COSs after 72 h of incubation.

**Figure 10:**
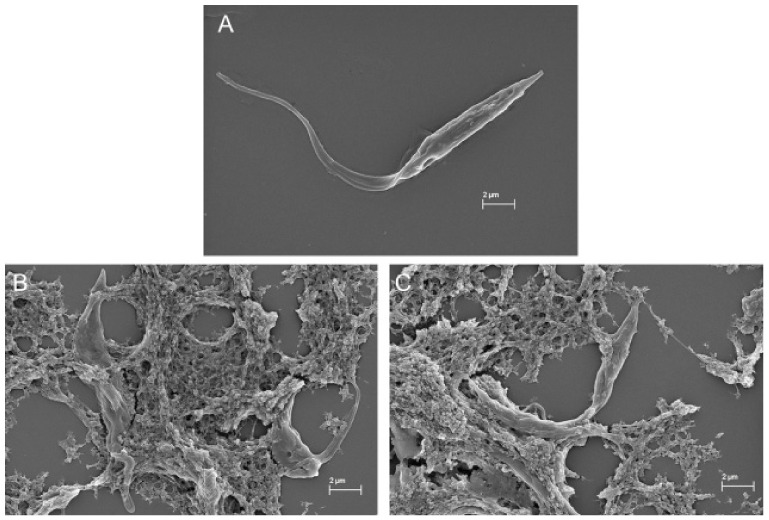
SEM images of (**A**) epimastigote forms of *T. cruzi*; (**B**,**C**) epimastigote treated with 400 μg/mL COSs after 144 h of incubation.

In the SEM images, trypanosomatids are observed as elongated cells with a thin singular flagellum and a smooth surface (Figure 9A and Figure 10A). After 72 h of incubation with COSs, morphological alterations such as rough surface and membrane disruption were seen in the promastigotes of *L. amazonensis* (Figure 9B,C). Additionally, the presence of a net that adheres to the parasites, forming aggregates, is noticed in Figure 9D. This same net is visible in epimastigote forms of *T. cruzi* treated with COSs (Figure 10B,C).

**Figure 11:**
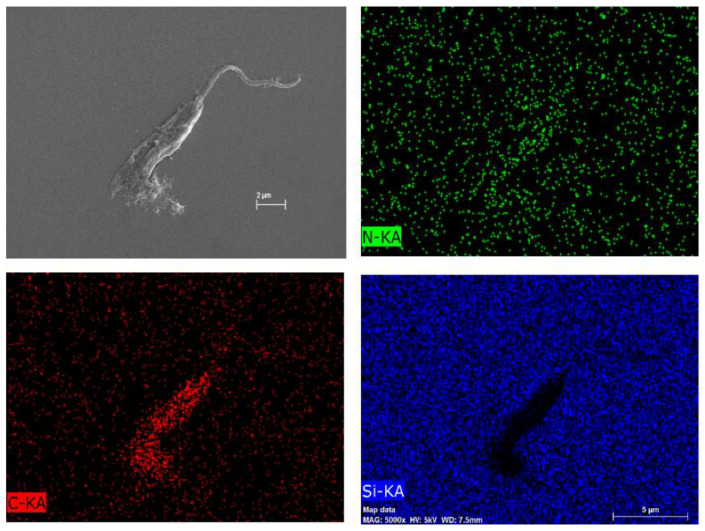
Atomic composition map by EDS of Figure 7 of *L. amazonensis* treated with COSs after 72 h. Blue spots indicate silicon atoms, red spots indicate carbon, and green spots indicate nitrogen.

To investigate the composition of this particular net, Figure 9B underwent an atomic composition analysis using the EDS technique (Figure 11). The presence of carbon and nitrogen concludes that the net is an organic material. Nonetheless, it is not possible to certify whether the net is either the COS or the extravasate of the internal contents of the parasites, but it is probably a combination of both oligomer and extravasate.

## 3. Discussion

Chitosan hydrolysis using chitosanases from *B. toyonensis* generated a biological active COS mixture in a very short reaction time. The structural characterization by NMR determined a deacetylation degree of 81.14% for the COS mixture, and the MS analysis indicated the presence of an acetylated hexamer within the mixture. This corroborates earlier studies that suggested that COSs with a DP of 5–6 are closely related to biological activity [41,42].

Apart from COSs, chitosan hydrolysis also produces GlcN and GlcNAc monomer units. Glucosamine was previously related to inducing cytotoxicity in a COS mixture [43]. Therefore, in order to investigate if the hydrolysis of chitosan would promote molecules with cell toxicity, the biocompatibility assay was performed. The resemblance of MTT reduction in chitosan and oligomers indicates the maintenance of cell viability, suggesting the absence of alterations in the cytocompatibility. The biocompatibility of chitosan, as well as its oligomers, has already been demonstrated [43,44]. Additionally, COS induced cell growth in RAW cells suggests a proliferative and a probable immunostimulant effect. The immunomodulation potential of COSs has been described as an anti-inflammatory property to inhibit IL-6, IL1β, and NO [45,46].

The antimicrobial evaluation of COSs was performed using Gram-positive and Gram-negative bacteria and *Candida* species. Between authors, there is no consensus on what type of bacteria COSs exert a higher effect. Li et al. demonstrated that COSs exert a higher effect upon Gram-positive than Gram-negative bacteria at the same pH; however, a DP ≥ 5 is essential for antibacterial activity [47]. In more recent work, COSs with an average MW of 17.2 kDa demonstrated a higher inhibition of Gram-negative bacteria [21]. Therefore, COSs’ MW, DD, and PA play major roles in antimicrobial activity characteristics [48,49].

In our results, the MIC for *S. aureus* (1 mg/mL) was lower than that defined for COSs with a MW < 5 kDa [50]. The MIC value found for COSs against *P. aeruginosa* was also lower than described for the conjugation of COSs with gold nanoparticles (≈4 mg/mL) [51]. It was not possible to determine the MIC for *E. coli* in our experiment since it was higher than 2 mg/mL; however, this is in agreement with the MIC value of 5 mg/mL described for *E. coli* treated with acetylated COSs with an average MW of 17.2 kDa [21]. Interestingly, hexamers had a better inhibitory effect upon *E. coli* among purified COSs, ranging from monomers to hexamers—once more demonstrating that our COS mixture has suitable structural characteristics of antimicrobial potential. The MIC value of COSs against *S. epidermidis* was also lower than 0.25 mg/mL, and thus not identified. The effect of COSs upon these bacteria is poorly investigated, with an MIC value of at least 0.6 mg/mL related to hetero-COS [52].

The interaction between COSs and the bacterial membrane leads to membrane disruption and consequent intracellular leakage [53]. This property was explored in the creation of silver nanoparticles functionalized with COSs to obtain antibacterial synergism [28]. COSs have greater solubility and a smaller size than chitosan, and they have also demonstrated a greater interaction capacity and ease of access in DPPC (dipalmitoyl phosphatidylcholine) and DPPG (dipalmitoyl phosphatidylglycerol) membranes, and the ability to penetrate the bacteria acting internally [54,55]. The advantage of COSs over chitosan can be seen in the practical example of the analysis of COS-Streptomycin conjugates against *Pseudomonas aeruginosa* biofilm [56].

*C. albincans* and *C. tropicalis* are medically important species of *Candida* regarding their virulence and current clinical resistance to commonly used antifungals, such as azoles [57]. COSs were active against these yeasts, with an MIC of 0.5 mg/mL for *C. albicans*, similar to that described for oligomers with 10 kDa (MIC of 512 μg/mL) [58], and a better activity against *C. albicans* and *C. tropicalis* than chitosan hydrogel [59]. COS activity against other fungal species is described elsewhere [60,61]. Concerning the lower growth inhibition of *C. albicans* and *C. tropicalis* treated with a higher concentration of COSs, this can be explained by the presumable COS self-aggregates formation, preventing the interaction of the carbohydrate with the bacteria [62].

The antifungal mechanism of COSs is similar to the antibacterial membrane disruption [63]; the SEM images also demonstrated membrane deformation and the suppression of hyphal formation [58]. The synergistic action of COSs with commercial antifungals used in human health and agriculture was formerly investigated and underlines the potential of these molecules in the combat against microbial resistance. Even when COSs did not impair cell growth when interacting with the yeast membrane, they were able to cause sufficient perturbations to potentialize the effect of Fluconazole in azole-resistant *Candida* [64,65].

Previous studies evaluating the antiprotozoal activity of COSs are scarce. Studies with GlcN and GlcNAc demonstrated that these monomers cause rapid growth on procyclic forms of *T. brucei*, followed by a decline in growth due to toxic or inhibitory effects after 5 days of incubation [66]. These results were similar to our observation on parasitic growth, indicated by the rise in the resazurin reduction after COS incubation, and a later inhibitory phase. GlcNAc cannot be consumed by this parasite, but it promotes effects such as a metabolic shift in *T. brucei* [67]. The probable action of GlcNAc is linked to the interactions with the membrane surface, such as the lecithin receptor [68], or by electrostatic interactions with the sialic acid on the parasitical membrane [69]. Chitosan is also used as a drug-carrier for Chagas disease [70,71].

The leishmanicidal activity was described for chitosan [72,73]. Recently, high molecular weight chitosan displayed a better inhibitory effect upon promastigote and amastigote forms of *L. major* and *L. mexicana* than medium and low molecular weight chitosan, as well as its oligomers. The suggested mechanism was the accumulation of chitosan on the parasitophorous vacuole acting directly on the parasite [33]. Different from the Trypanosome sp., the genus Leishmania can catabolize GlcNAc, and to a lesser degree GlcN, in the interior of macrophages. Thus, the accumulation of hexosamine-phosphate could lead to toxicity in the parasite [74].

Consequently, our research suggests that COSs can form a polymeric net that interacts with the parasite, either preventing the locomotion and probably the nutrient exchanges of the parasite through membrane receptors or disturbing the parasitic metabolism. After 72 h of incubation, toxic COS activity occurs upon the promastigote forms of *L. amazonensis*, leading to morphological alterations, as seen in the SEM images. Even though the morphological effects of COSs upon epimastigote forms of *T. cruzi* were not evident in the SEM images, the oligomers also caused time-dependent inhibition of these parasites. A similar feature of COSs forming aggregates with bacteria, which could interfere in the nutrient exchange, was mentioned earlier [47,75]. Even if these interactions did not cause parasite death, it could make them more sensible, as it was discussed in the antifungal mechanism [65].

Therefore, COSs’ characteristics of biocompatibility, higher water solubility, and adherence visualized by a net in the SEM images, together with their broad antimicrobial effect and demonstrated here for the first time for anti-*Trypanosoma cruzi* activity, reinforce the use of COSs as a versatile biomaterial in health. The possibility of enhancing their pharmacological activity with a synergistic effect when combined with drugs, or in a drug delivery system, is a trend to overcome bacterial resistance, and it can be a strategy, as well, in the treatment of leishmaniasis and Chagas disease. Thus, this study expands the opportunities of COSs’ application in biomedical science by demonstrating their antiprotozoal activity.

## 4. Materials and Methods

### 4.1. Materials

Low molecular weight chitosan (DD 85%) and 3-[4,5-dimethyl-thiazol-2-yl]-2,5-diphenyltetrazolium bromide (MTT) were obtained from Sigma-Aldrich Co (Saint-Louis, MO, USA). The bicinchoninic Acid (BCA)Protein Assay kit was purchased from Thermo Fischer Scientific (Waltham, MA, USA). A Hiprep 16/60 Sephacryl S-100 HR column was purchased from GE Healthcare Bio-science (Uppsala, Sweden), and a LUNA phenyl-hexyl column 250 × 4.6 × 5 μm was purchased from Phenomenex (Torrance, CA, USA). All other chemical reagents used in this study were purchased commercially and were of adequate analytical grade. *B. toyonensis* were provided by the Biochemistry Engineering Laboratory from the Federal University of Rio Grande do Norte (UFRN), under the following registration number at the National System of Genetic Resource Management and Associated Traditional Knowledge (SisGen): AD8AE98/Nov 2018.

### 4.2. Chitosanase Production

COSs were produced by enzymatic hydrolysis using chitosanases obtained by the cultivation of *Bacillus toyonensis* CCT 7899. This strain was selected due to its production of a stable chitosanase with optimum conditions at 55 °C and pH 6.0 [32,34]. Bacteria were grown in liquid media, as previously described [34]. The broth was collected and centrifugated to obtain the enzymatic extract. Enzymatic activity was measured using the dinitrosalicylic acid method for quantification of reducing sugar [76], and total protein was measured using the bicinchoninic acid method, using the BCA Protein Assay kit (Thermofisher Scientific).

### 4.3. COS Production

A total of 2.5 mg of enzymatic extract, containing chitosanases, was added to 5 mL of a solution of chitosan 1% (*w*/*v*) at pH 6.0. The hydrolysis was carried out in a water-bath at 55 °C for 10 min, and it was stopped by placing the reaction tubes in boiling water for 10 min [34]. The hydrolysate was centrifugated at 3062× *g* for 20 min at 24 °C. To the supernatant, ethanol 99% (*v*/*v*) was added and incubated at 4 °C overnight in order to precipitate the COSs. The precipitate was collected by centrifugation and lyophilized (COS) [77]. For performance assays, the samples were prepared as follows: COSs were solubilized in acid water and chitosan (CHI) was solubilized in HCl 0.1N, and the final pH of all the samples was 6.0 ± 0.5. Acid water was obtained by adjusting the pH of purified water with a few microliters of an HCl 6N solution. The final pH was adjusted using a diluted solution of HCl or NaOH.

### 4.4. COS Characterization

The oligomers were characterized using the Nuclear Magnetic Resonance (NMR) technique with an NMR spectrometer (AVANCE III HD NMR SPECT 300 MHz, BRUKER, Billerica, MA, USA). ^1^H and ^13^C one-dimensional and COSY, HSQC, and HMBC two-dimensional spectra were obtained. Samples were dissolved in deuterated water. The deacetylation degree of COSs was determined according to Equation (1), where A1 corresponds to the average area of the protons within the sugar ring C_2_-C_6_ (3–6 ppm) and A2 corresponds to the average area of the CH_3_ protons from the GlcNAc unit (2 ppm) [35]:

DD(%) = [1 − ((6 × A2)/(3 × A1))] × 100,(1)

Prior to the mass spectrometry analysis, COSs (10 mg/mL) were injected into a Hiprep 16/60 Sephacryl S-100 HR column in an AKTÄ system (GE Healthcare Chicago, IL, USA, with eluent flow consisting of acetate buffer (0.1 M, pH 6.0) at a flowrate of 1 mL/min. The fractions were analyzed with an HPLC-ESI-QToF-MS/MS. A LUNA phenyl-hexyl column 250 × 4.6 × 5 μm was used with 1 mL/min flow rate, an oven temperature of 30 °C, an injection volume of 40 μL, and a mobile phase consisting of formic acid 0.1% (*v*/*v*) (A) and formic acid in acetonitrile 0.1% (B). The gradient started at 2% (*v*/*v*) B for 10 min, followed by raising from 2% to 100% B in 20 min, keeping at 100% B for 5 min, returning to the initial conditions in 5 min, and keeping at 2% B during the last 5 min. The mass spectrometer was employed using scan mode on ions between *m*/*z* 50 and 1500 and capillary 3.5 KV. The fragmentation was set to automatic mode to the 5 most intense ions by each cycle of 3 s and the collision energy varied for each ion. The drying gas was nitrogen and was set to 220 °C, 9 L/min, and 4.5 bar. Sodium trifluoroacetate 4 mg/mL was used as an internal and external calibrator.

### 4.5. Biocompatibility Assay

Kidney epithelial cells from the African green monkey (Vero E6, ATCC CRL-1586) and murine macrophages (RAW 264.7, ATCC TIB-71) were kindly supplied by the Laboratory of Biotechnology of Natural Polymers at UFRN. Cells were grown in Dulbecco’s Modified Eagle Medium (DMEM) supplemented with 10% (*v*/*v*) fetal bovine serum (FBS)in 96-well plates for 24 h at 37 °C and 5% CO2 until confluent. Increasing concentrations of CHI or COSs (0.625–2 mg/mL) were added to the cells and incubated for 24 h at 37 °C and 5% CO2 [78]. Then, a solution of 2 mg/mL of 3-[4,5-dimethyl-thiazol-2-yl]-2,5-diphenyltetrazolium bromide was added to the plates and incubated for 4 h [79]. Alcohol 96% was used to solubilize the formazan crystals, and absorbance was measured at 570 nm in a microplate reader (Epoch-Biotek^®^, Winooski, VT, USA). Cells incubated in the absence of the polymers were used as positive controls; cell viability was calculated based on the positive control (100% cell growth) and expressed as a percentage of MTT reduction.

### 4.6. In Vitro Antimicrobial Activity of COSs

The microorganisms Escherichia coli (ATCC 25922), Pseudomonas aeruginosa (ATCC 27853), Klebsiella pneumoniae (ATCC 10031), Staphylococcus aureus (ATCC 29213), Staphylococcus epidermidis (ATCC 12228), Enterococcus faecalis (ATCC 29212), Candida albicans (ATCC 90028), and Candida tropicalis (ATCC 13803) were obtained at the Clinical Microbiology Laboratory at UFRN and maintained in nutrient agar at 4 °C. Antimicrobial assays were performed using the microdilution method in Mueller Hinton broth (MHB); inoculums of 10^5^ CFU/mL for bacteria and 10^4^ CFU/mL for yeast were prepared as mentioned in the Clinical and Laboratory Standards Institute (CLSI) guidelines [80,81]. COSs (0.25–2 mg/mL) were added in 96 well-plates together with the inoculum in MHB. Then, plates were incubated at 35 ± 2 °C at 200 rpm for 24 or 48 h. Microbial growth was measured by the optical density at 595 nm in a microplate reader (Epoch Biotek, Winooski, VT, USA). Wells containing only microorganism suspensions or sterile saline solution 0.9% (*w*/*v*) were used as a positive and negative control of growth, respectively. The minimal inhibitory concentration (MIC) was defined as the lowest concentration of the sample capable of inhibiting the visible growth of the microorganism.

### 4.7. In Vitro Antiparasitic Activity of COSs

#### 4.7.1. Leishmania Amazonensis

Promastigote forms of *L. amazonensis* were cultivated in RPMI 1640 at 27 ± 2 °C for 4 days until the log phase. A volume of 200 μL of a parasitic inoculum (10^7^ parasites/mL) was added to COSs, at concentrations of 25 to 400 μg/mL in a 96 well-plate, and incubated for 24, 48, or 72 h at 27 ± 2 °C. Parasitic viability was measured by the resazurin reduction assay. Briefly, 20 μL of 1 mM of resazurin was added to the plates after each incubation time and then incubated again for 24 h. Absorbance was determined at 570 and 600 nm in a microplate reader (Epoch Biotek, Winooski, VT, USA). The percentage of inhibition was calculated using Equation (2):

% Inibition = 100 − ((A570t − (A600t × R0))/(A570c − (A600c × R0))) × 100,(2)

A570t: Absorbance of the treatment at 570 nm; A600t: Absorbance of the treatment at 600 nm; A570c: Absorbance of the control at 570 nm; A600c: Absorbance of the control at 600 nm. R0: Correction factor of the influence of the media on the resazurin reduction, the product of absorbance of the media at 570 nm to the absorbance of the media at 600 nm [82].

#### 4.7.2. Trypanosoma Cruzi

Epimastigote forms of *T. cruzi* were cultivated in LIT medium (Liver Infusion Triptose) at 27 ± 2 °C for 5 to 7 days until the log phase. A volume of 200 μL of a parasitic inoculum (10^7^ parasites/mL) was added to a 96 well-plate with COSs, at concentrations of 400 to 25 μg/mL, and incubated for 24, 48, 72, or 144 h at 27 ± 2 °C. The parasitic viability was measured by the resazurin reduction assay, as mentioned in 4.7.1. 

#### 4.7.3. Morphological Analysis Using Scanning Electron Microscopy

The morphology of epimastigote and promastigote forms of *T. cruzi* and *L. amazonensis*, respectively, was visualized using scanning electron microscopy SEM-FEG ZEISS AURIGA 40 (Zeiss, Oberkochen, Germany). A concentration of 400 μg/mL of COSs was added to the parasites (1 × 10^7^ parasites/mL) and incubated for 72 or 144 h at 27 °C. After centrifugation at 194× *g*, 4 °C for 10 min, the protozoa pellet was washed twice using saline solution 0.9% (*w*/*v*). Cells were fixed with 2.5% glutaraldehyde in saline solution 0.9% (*w*/*v*) at 4 °C for 4 h, followed by dehydration using increasing concentrations of ethyl alcohol. Parasite samples were sent to the Structural Characterization Laboratory of Materials of the Federal University of Rio Grande do Norte (Natal, Brazil) for imaging [83].

### 4.8. Statistical Analysis

Data are expressed as mean ± standard deviation. Statistical analysis was performed using one-way analysis of variance (ANOVA) followed by Tukey’s test, with GraphPad Prism software (version 7.00, GraphPad, San Diego, CA, USA). Data were considered significant when the p-value was less than 0.05 (*p* < 0.05).

## 5. Conclusions

The biotechnological process involved in generating biological macromolecules from seafood waste represents an advance in the green economy. Numerous studies have been establishing the versatile use of COSs, especially in the biomedical area. In this scenario, our study places itself by corroborating with previous investigations and adding a new opportunity to explore the antiprotozoal effect of COSs, mainly against NTDs. The hydrolysis of chitosan using the enzymatic extract obtained by *B. toyonensis* produced COSs with biological activities in a short hydrolysis time. COSs did not produce cytotoxic effects on normal cells and also exerted broad antimicrobial activity. The antiparasitic effect of COSs was time-dependent, and they were also able to induce parasite aggregation and membrane alterations. Additionally, further studies of the effect of COSs on these parasites in vitro and in vivo are still needed to better elucidate the action mechanism, enabling their application in a pharmaceutical formulation as well as the clinical use of these oligomers.

## Figures and Tables

**Table 1:** Minimal inhibitory concentration (MIC) values of COSs upon different microorganisms.

Microorganism	MIC
*K. pneumoniae* (ATCC 10031)	>2 mg/mL
*P. aeruginosa* (ATCC 27853)	1 mg/mL
*E. coli* (ATCC 25922)	>2 mg/mL
*S. epidermidis* (ATCC 12228)	<0.25 mg/mL
*E. faecalis* (ATCC 29212)	2 mg/mL
*S. aureus* (ATCC 29213)	1 mg/mL
*C. albicans* (ATCC 90028)	0.5 mg/mL
*C. tropicalis* (ATCC 13803)	1 mg/mL

## Data Availability

Data is contained within the article or Supplementary Material.

## Supplementary Materials

The following are available online at https://www.mdpi.com/1660-3397/19/2/110/s1. Figure S1: Chromatogram HPLC-MS/MS of COS samples between 1.5 to 4 min run. Orange: Chitosan; dark green: COSG1; green: COSG2; light green: COSG3; Figure S2: A. HPLC-MS/MS chromatogram from 19.2 to 20.1 min of the fractions COSG1 (dark blue), COSG2 (Blue), and COSG3 (light blue); B. MS spectrum to the peak in 19.4 min from COSG3; C. MS spectrum to the peak in 19.6 min from COSG3; D. MS spectrum to the peak in 19.8 min from COSG3.
