# Supplementary material for: Antimicrobial Activity of Chitosan Oligosaccharides with Special Attention to Antiparasitic Potential
Source: Mar Drugs. 2021 Feb 12;19(2):110. doi: 10.3390/md19020110 (PMC7917997; doi:10.3390/md19020110)
Supplement: Supplementary file 1 [file marinedrugs-19-00110-s001.pdf]

Figure S1: Chromatogram HPLC-MS/MS of COS samples between 1.5 to 4 min run. Orange: Chitosan; dark green: COSG1; green: COSG2; light green: COSG3.

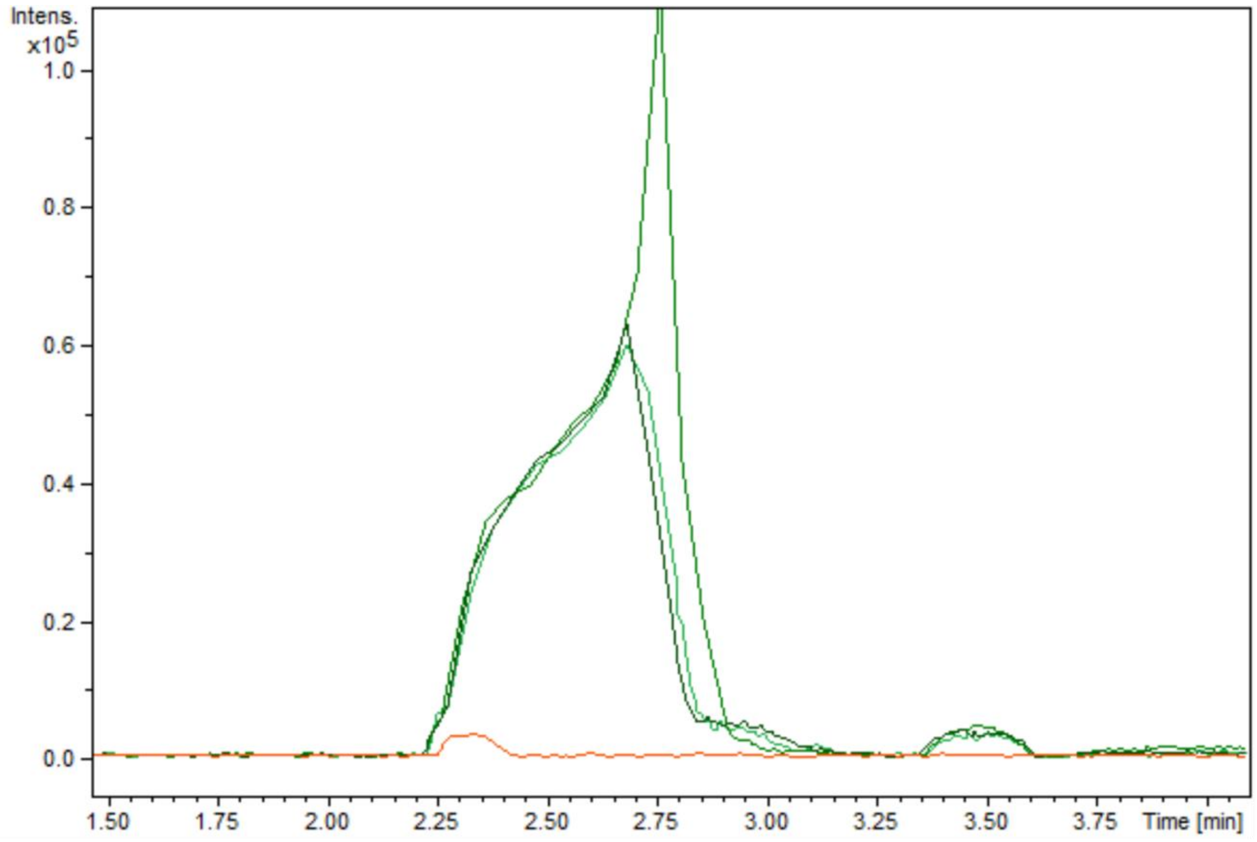

Figure S2: A. HPLC-MS/MS chromatogram from 19.2 to 20.1 min of the fractions COSG1 (dark blue), COSG2 (Blue), and COSG3 (light blue); B. MS spectrum to the peak in 19.4 min from COSG3; C. MS spectrum to the peak in 19.6 min from COSG3; D. MS spectrum to the peak in 19.8 min from COSG3.

A

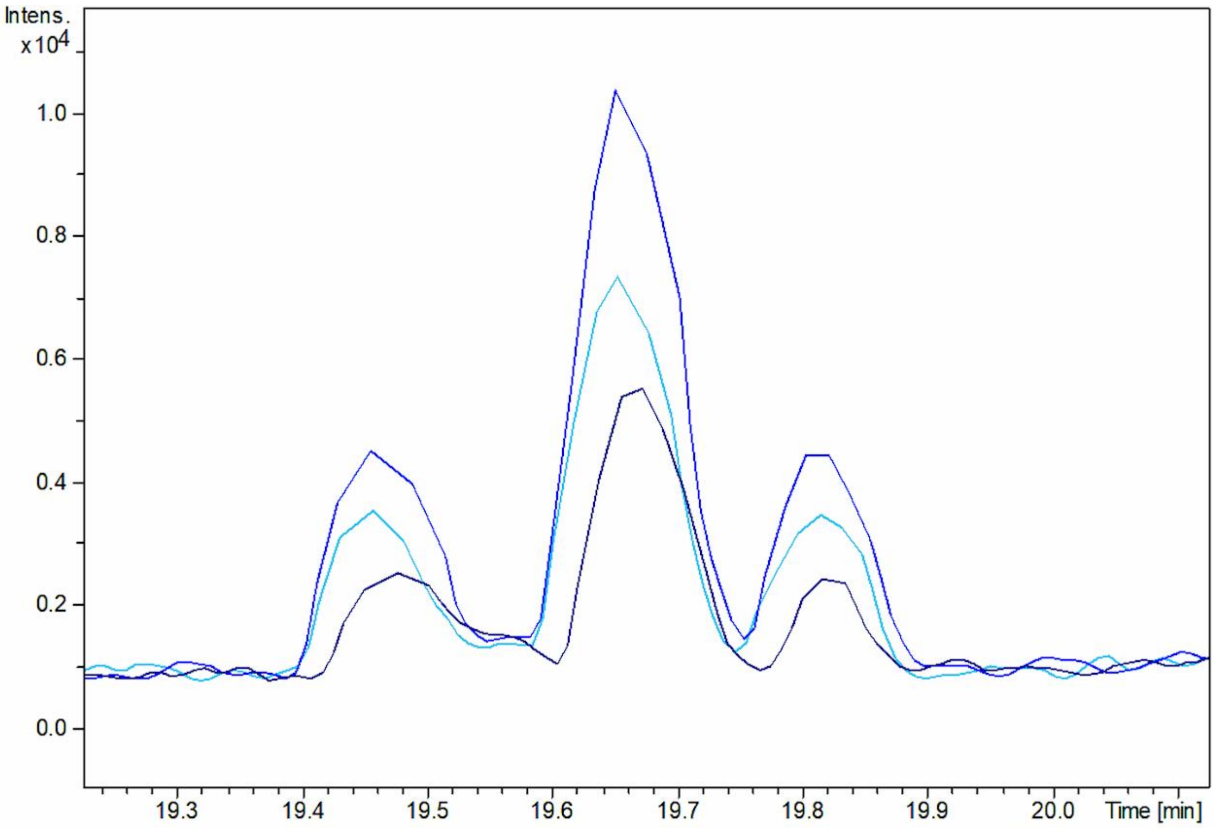

B

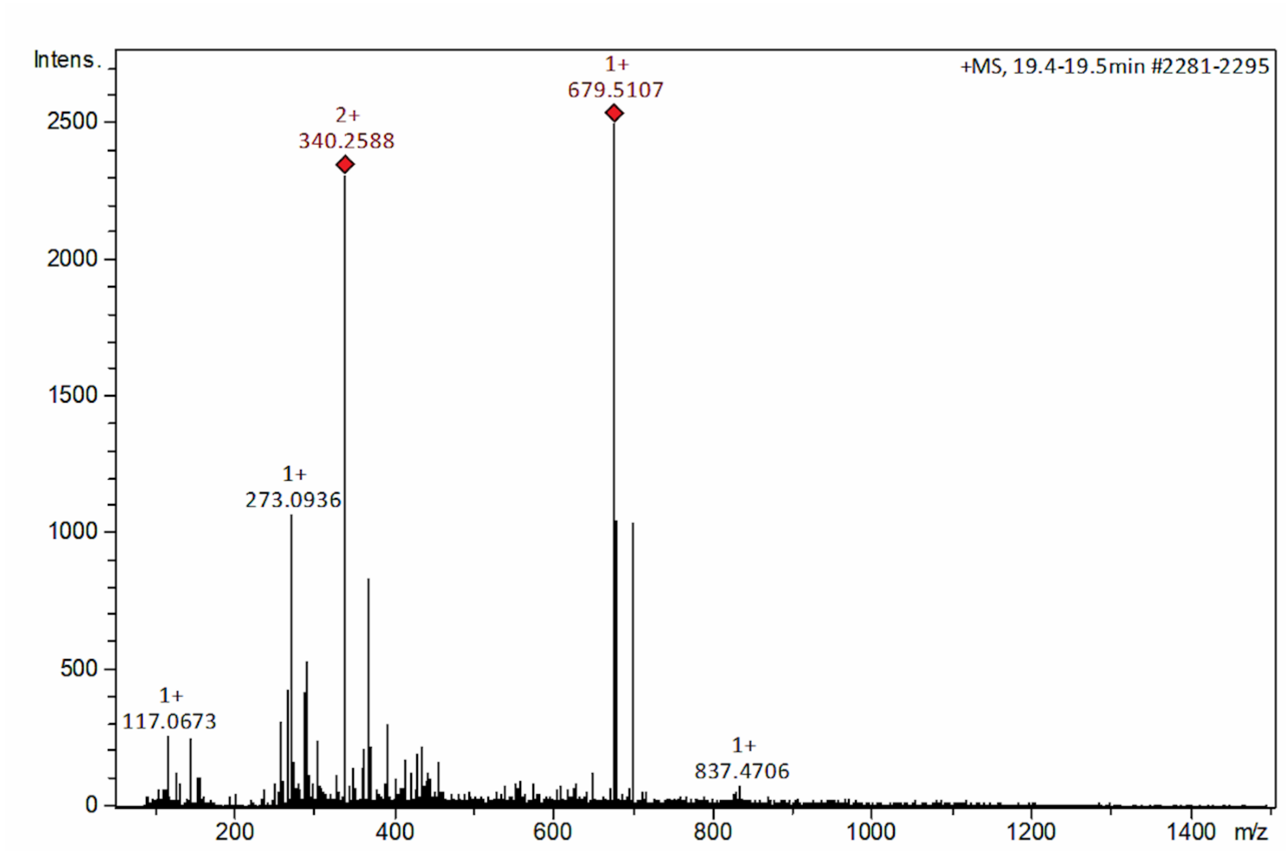
Intens.
+MS, 19.4-19.5min #2281-2295
1+
679.5107
2+
340.2588
1+
273.0936
1+
117.0673
1+
837.4706
2500
2000
1500
1000
500
0
200
400
600
800
1000
1200
1400
m/z

C

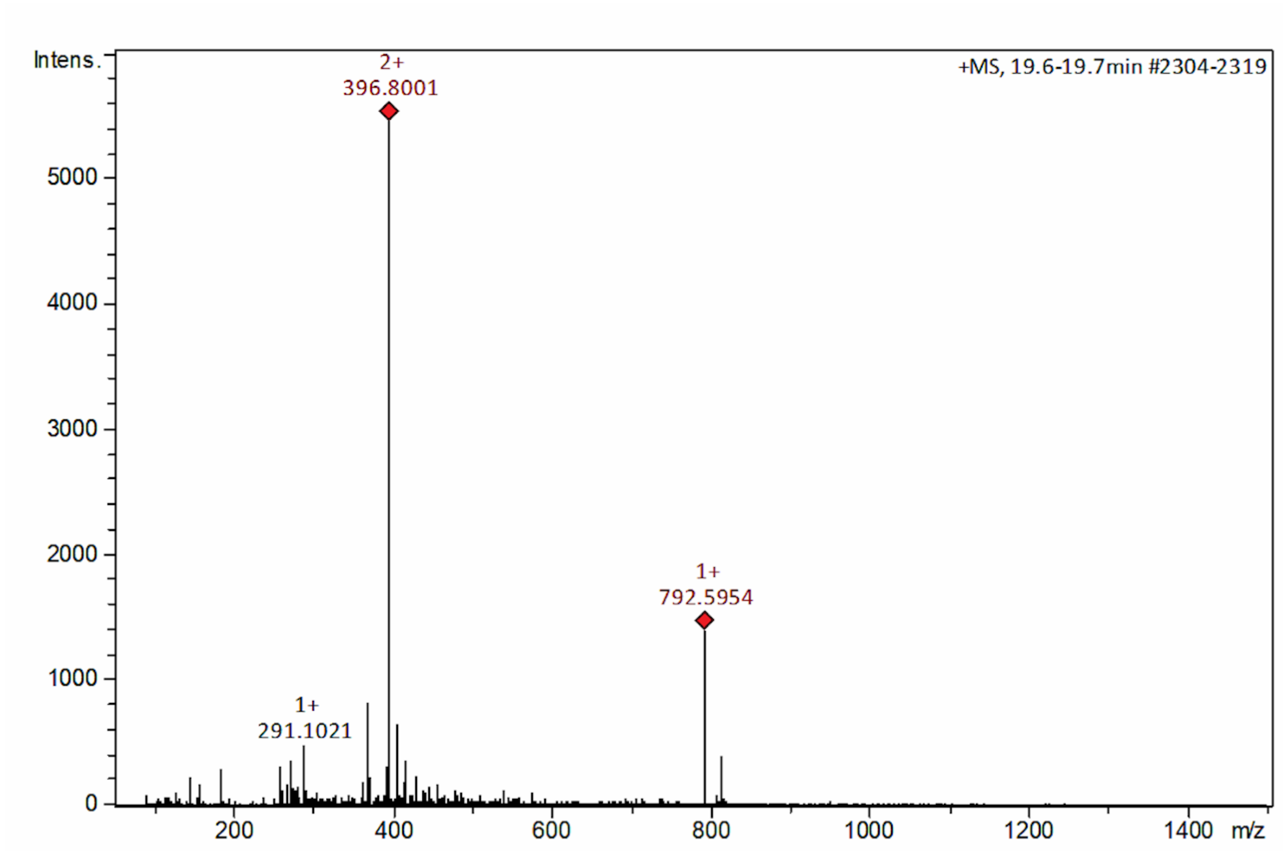
Intens.
+MS, 19.6-19.7min #2304-2319
2+
396.8001
1+
792.5954
1+
291.1021
5000
4000
3000
2000
1000
0
200
400
600
800
1000
1200
1400
m/z

D

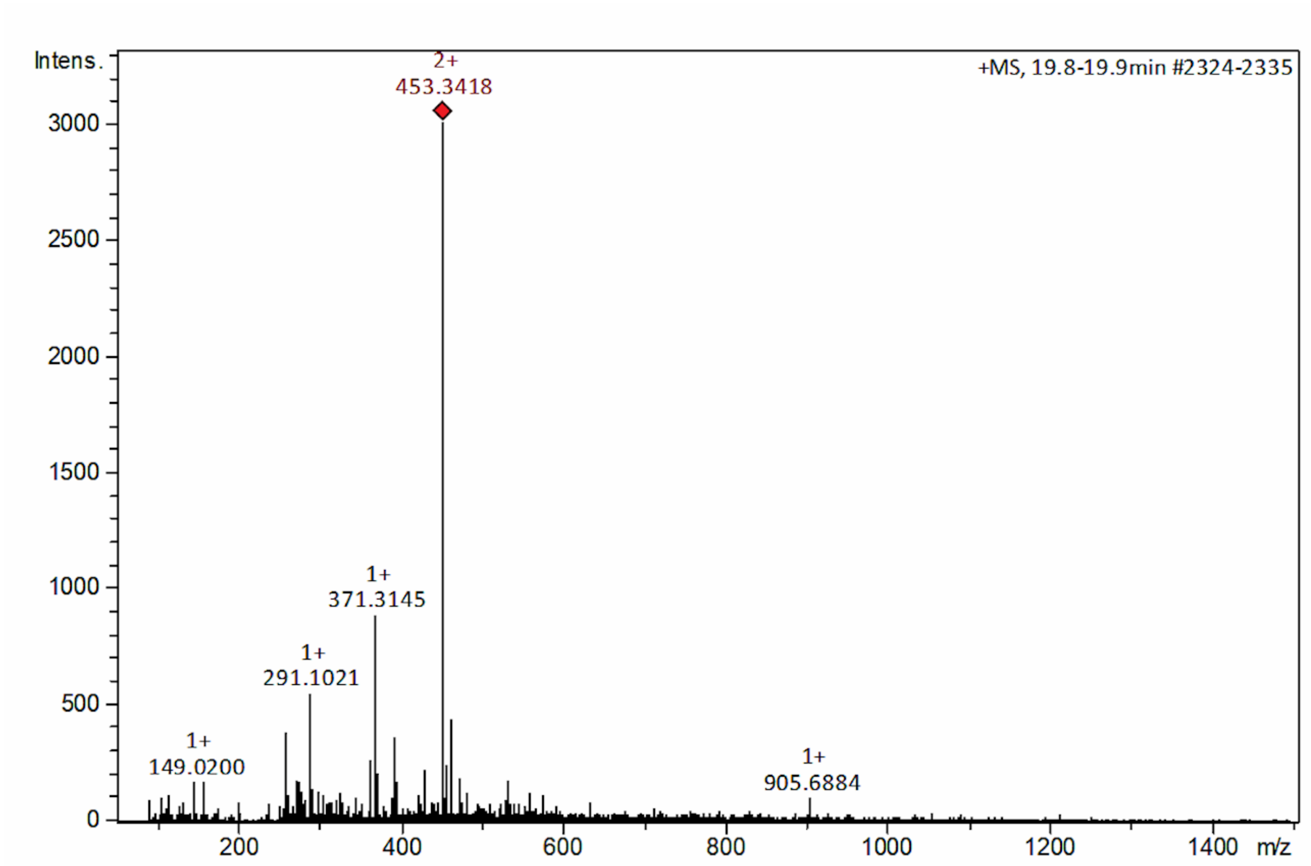
Intens.
+MS, 19.8-19.9min #2324-2335
2+
453.3418
1+
371.3145
1+
291.1021
1+
149.0200
1+
905.6884
3000
2500
2000
1500
1000
500
0
200
400
600
800
1000
1200
1400
m/z
